# De novo regulation of RD3 synthesis in residual neuroblastoma cells after intensive multi-modal clinical therapy harmonizes disease evolution

**DOI:** 10.1038/s41598-019-48034-2

**Published:** 2019-08-13

**Authors:** Dinesh Babu Somasundaram, Karthikeyan Subramanian, Sheeja Aravindan, Zhongxin Yu, Mohan Natarajan, Terence Herman, Natarajan Aravindan

**Affiliations:** 1 Department of Radiation Oncology, University of Oklahoma Health Sciences Center, Oklahoma City, OK USA; 2 Stephenson Cancer Center, Oklahoma City, OK USA; 3 Department of Pathology, University of Oklahoma Health Sciences Center, Oklahoma City, OK USA; 4 Department of Pathology, University of Texas Health Sciences Center, San Antonio, TX USA

**Keywords:** Metastasis, Paediatric cancer

## Abstract

Most high-risk neuroblastomas that initially respond to therapy will ultimately relapse. Currently, no curative treatment is available. Acquired genetic/molecular rearrangement in therapy-resistant cells contributes to tumor relapse. Recently, we identified significant RD3 loss in progressive disease (PD) and defined its association with advanced disease-stage and poor clinical outcomes. Here, we investigated whether RD3 loss is an acquired process in cells that survive intensive multi-modal clinical therapy (IMCT) and its significance in disease evolution. RD3 status (mRNA, protein) during diagnosis (Dx) and PD after IMCT was investigated in NB patient cohort (*n* = 106), stage-4 NB cell lines (*n* = 15) with known treatment status and validated with independent data from another set of 15 cell-lines. Loss of RD3 in metastatic disease was examined using a mouse model of PD and metastatic-site-derived aggressive cells (MSDACs) *ex vivo*. RD3 silencing/expression assessed changes in metastatic state. Influence of RD3 loss in therapy resistance was examined through independent *in vitro* and *in vivo* studies. A significant loss of RD3 mRNA and protein was observed in resistant cells derived from patients with PD after IMCT. This is true to the effect within and between patients. Results from the mouse model identified significant transcriptional/translational loss of RD3 in metastatic tumors and MSDACs. RD3 re-expression in MSDACs and silencing RD3 in parental cells defined the functional relevance of RD3-loss in PD pathogenesis. Analysis of independent studies with salvage therapeutic agents affirmed RD3 loss in surviving resistant cells and residual tumors. The profound reductions in RD3 transcription indicate the de novo regulation of RD3 synthesis in resistant cells after IMCT. Defining RD3 loss in PD and the benefit of targeted reinforcement could improve salvage therapy for progressive neuroblastoma.

## Introduction

Neuroblastoma (NB) accounts for nearly one tenth of all pediatric cancer deaths^1,2^. Despite intensive multi-modal clinical therapy (IMCT)^1,3–7^ more than 50% of patients with high-risk phenotypes will relapse with hematogenous metastasis^8^. Given the disease’s heterogeneity, resistance, and poor hematological reserve, cure of high-risk disease is rare, with <10% 5-year overall survival (OS) and 2% 10-year survival, compared with 38–71% for low-risk disease^7,9^. High-risk disease is typically characterized by a variety of genetic and molecular rearrangements^10,11^. Somatic amplification of MYCN in about 20% of NB patients is independently associated with advanced stage and poor clinical outcomes^12–14^. However, MYCN amplification is restricted to about 25–35% of the high-risk phenotype, while the remaining 65–75% of progressive NB is MYCN non-amplified (MYCN-na)^15–17^. The IMCT for high-risk NB comprises *induction phase* with alternating regimens of high-dose chemotherapeutic drugs and load reduction surgery; *consolidation phase* with more intensive chemotherapy along with radiotherapy and stem cell transplant, and; *maintenance phase* with retinoid drug treatment, immunotherapy, and immune-activating cytokine treatment. Despite such intensive treatment, high-risk MYCN-na patients have only 37% 5-year OS and 9% 10-year OS^18,19^. Identifying the crucial molecular targets, defining their orchestration, and understanding the signal-transduction flow-through that drives MYCN-na progressive disease (PD) could lead to the development of an efficient and improved therapeutic strategy and better patient outcomes.

The relapse timeline of >18 months for the first recurrence and decreasing rapidly thereafter^5,20^ reflects acquisition of genetic and molecular rearrangements in the undifferentiated tumorigenic neural crest cells that mediate NB progression^21–23^. Our recent investigations using a mouse model of PD indicated that aggressive CSC-like NB cells exhibit reversible and adaptive plasticity, which could determine the evolution of NB^24^. High-throughput (miRNA, cGH) characterization of this model recognized acquisition of genetic/molecular rearrangements in disease evolution^25–27^. We demonstrated that Retinal Degeneration Protein 3 (RD3), which is constitutively expressed in human tissues^28^, has a regulatory role in NB evolution, and RD3 loss (i) contributes to the altered metastatic state of the NB cells *in vitro* and (ii) pathogenesis of disease progression *in vivo*, and (iii) is associated with advanced disease stage and poor clinical outcomes^29^.

In the present study, we used bio specimens from a cohort of NB patients, a panel of stage 4 MYCN-na human cell lines coupled with appropriate *in vitro*, *in vivo*, and *ex vivo* NB models to investigate molecular alterations in MYCN-na NB cells that could lead to significant improvements in IMCT. We focused on defining the acquisition of RD3 loss with IMCT and any association of RD3 loss with disease evolution and clinical outcomes. We investigated the transcriptional (mRNA) and translational status of RD3 in 15 high-risk stage 4 MYCN-na NB cell lines, before and/or after IMCT, and recognized the association of RD3 with disease evolution. Using *in silico* data analysis, we investigated the association of RD3 loss with patient outcomes in MYCN-na NB cohorts.

## Methods

### Cell culture

Fifteen high-risk NB stage-4 MYCN-na cell lines (CHLA-61, CHLA-171, CHLA-40, CHLA-172, CHLA-15, LA-N-6, COG-N-291, SK-N-FI, CHLA-42, CHLA-20, CHLA-90, CHLA-79, NB-EBc1, SMS-LHN, and CHLA 60) were obtained from the COG-NB cell repository. The details, including patient gender, age, disease stage, MYCN status, phase of therapy, source of culture, and IMCT, are provided in Table S1. In-house culture and maintenance of CHLA-61, CHLA-171, CHLA-40, CHLA-172, CHLA-15, COG-N-291, CHLA-42, CHLA-20, CHLA-90, CHLA-79, NB-EBc1, and CHLA 60 was performed using IMDM supplemented with 20% FBS, 4 mM L-Glutamine, 5 μg/mL insulin, 5 μg/mL transferrin, 5 ng/mL selenous acid, and Pen-Strep (Penicillin, 12 units/mL; streptomycin, 12 µg/mL). LA-N-6, SMS-LHN, and SK-N-FI cells were cultured and maintained in RPMI-1640 medium supplemented with 10% FBS, 2 mM L-Glutamine, and Pen-Strep. All cell lines were authenticated by COG and are available online (http://www.cogcell.org/clid.php). The SK-N-AS cell line obtained from ATCC was cultured/maintained in DMEM, supplemented with 0.1 mM NEAA, 10% FBS, and Pen-Strep. For passaging and for all experiments, the cells were detached using 0.25% trypsin/1% EDTA, re-suspended in complete medium, counted (Countess), and incubated in a 95% air/5% CO_2_ humidified incubator.

### Cell-microarray construction and RNA *in situ* hybridization

**Figure 1:**
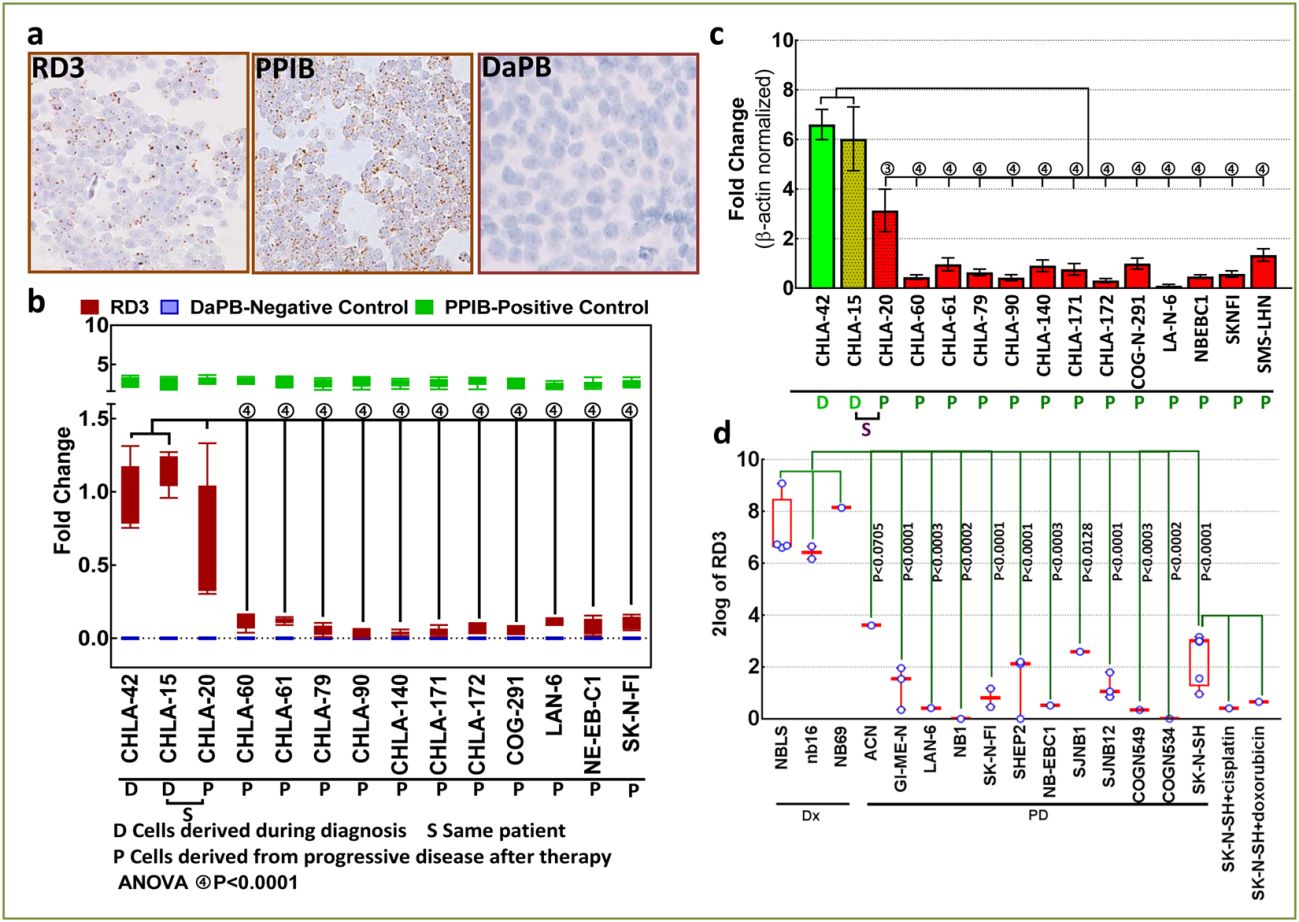
Transcriptional loss of RD3 with IMCT: (**a**) Representative microphotograph from RNAscope assay showing expression of RD3. All assays were performed on the custom-archived cell microarray with triplicate cores representing each cell line. Parallel PPIB and DaPB were used as positive and negative controls. (**b**) Box and whiskers plot from RNAscope analysis showing RD3 mRNA levels in MYCN-na neuroblastoma cell lines derived from stage 4 patients during diagnosis or after IMCT. Quantified levels of positive control PPIB and negative controls DapB are plotted in parallel. Compared with cell lines from diagnosis (CHLA-42 and CHLA-15), RD3 levels were significantly reduced in cell lines derived from progressive disease (PD) after IMCT. (**c**) Histograms from QPCR analysis showing a significant decrease in RD3 mRNA levels in cell lines derived from PD after IMCT compared with those derived during diagnosis. (**d**) Data mining from publicly available independent gene profiling studies showing significant reduction of RD3 transcription (RNA-seq data) in all cell lines derived after IMCT compared with the cell lines derived during diagnosis.

The cell microarray (CMA) approach allows us to measure RD3 levels across the 14 custom-archived MYCN-na cell lines, without inter-sample assay discrepancies. CMA construction and sectioning were performed in our Tissue-Pathology Core following standard protocols. Triplicate cores per cell line were assembled in a CMA block. *In situ* hybridization (ISH) for RD3 mRNA was performed using the RNAscope®2.5 HD-Detection Reagent – BROWN FFPE assay kit (ACD, Hayward, CA) according to the manufacturer’s instructions with custom target probes for human RD3, the housekeeping gene PPIB (positive control), or DapB (negative control) (Fig. 1a). RD3 mRNA expression profiles were quantified using NIH ImageJ, plotted with GraphPad Prism, and compared between groups using ANOVA with Tukey’s post-hoc correction.

### RD3 RNA ISH and immunohistochemistry (IHC) in clinical bio-specimens

We examined specimens from 106 cases human (MYCN-na, *n* = 83; MYCN amplified [MYCN-a], *n* = 23) NB, collected from our institutional (OUHSC) pediatrics pathology collection, Cooperative Human Tissue Network (CHTN) and the Oregon Health and Science University Bio-specimen core. All protocols were approved by the OUHSC Institutional Review Board (IRB), with permission for the research use of de-identified specimens. All experiments were performed in accordance with OUHSC IRB guidelines and regulations for the protection of human subjects. H&E-stained sections were examined, and only the cases with sufficient percent tumor (with minimal necrosis) and adequate tumor volume were included. RNA ISH for RD3 was performed for 92 cases (MYCN-na, *n* = 72 [specimens procured during Dx, *n* = 44; procured from PD after IMCT, *n* = 41; Dx and PD specimens from same patient, *n* = 13]; MYCN-a, *n* = 20 [Dx, *n* = 9; PD, *n* = 15; Dx/PD from same patient, *n* = 4] as discussed above. RD3 profiles were independently graded by three investigators in blinded fashion. IHC was performed for 72 cases (MYCN-na, *n* = 54 [Dx, *n* = 38; PD, *n* = 23; Dx/PD from same patient, *n* = 7]; MYCN-a, *n* = 18 [Dx, *n* = 10; PD, *n* = 13; Dx/PD from same patient, *n* = 5] in the Tissue Pathology Core of the Stephenson Cancer Center, as described in our earlier studies^29^. Appropriate tissue histology controls (H&E) and negative controls with no primary antibody were examined in parallel. The slides were digitally scanned into virtual slides using an Aperio ScanScope (Aperio Technologies, Inc., Buffalo Grove, IL, USA) slide scanner at 20× magnification. The whole slide images were then group-analyzed for RD3-specific positivity using Aperio image analysis and quantification software (Aperial Tool Box) with the appropriate algorithms for IHC. Two investigators also independently graded RD3 expression.

### Development of PD mouse model

All animal experiments conformed to American Physiological Society standards for animal care and were approved by University of Oklahoma Health Sciences Center Institutional animal care and use committee. SK-N-AS is an MYCN-na cell line, established from bone marrow metastasis of a poorly differentiated embryonal NB, and is composed of substrate-adherent neuroblasts. PD development in nude mice, assessment of tumor growth, and dissemination to distant sites were investigated as in earlier studies^26^. Animals were euthanized by CO_2_ asphyxiation. Cell derivations from tumors of primary/metastatic sites and *ex vivo* maintenance under stem cell culture conditions were performed as discussed earlier^24^. To reproduce PD, animals were injected with isolated and characterized clones of metastatic site-derived aggressive cells (SK-MSDACs).

### Tissue microarray (TMA) construction and TMA and CMA IHC

All TMA construction, RD3 IHC, and positivity scoring was performed and quantified as discussed in our earlier studies^27,28^. Dissemination of tumors to vital organs in PD were assessed by a pathologist (ZY) utilizing H&E- and synaptophysin (marker for NB) -stained sections. Similarly, a CMA with 14 MYCN-na cell lines was immunostained for RD3, scanned using Aperio, and analyzed with Spectrum. The TMA and CMA images were then group-analyzed for RD3-specific positivity using Aperio image analysis and quantification with the appropriate algorithms for TMA.

### Molecular assays

QPCR^29^, immunoblotting^30^, and high-content confocal immunofluorescence^27,29^ were performed as described earlier. For RD3 expression and silencing studies, the plasmid preparation and DNA/shRNA transfection were carried out as described earlier^29^. The cell migration was examined by both conventional scratch-wound (ibidi® insertions) and real-time wound healing (ORIS™) assays^29,31^. Cell invasion was examined using Matrigel invasion assay, and tumorosphere formation capacity was assessed using limiting dilution tumorosphere formation assay (LDTA), as described earlier^25,29^.

### *In silico* data analysis

We examined the correlation of RD3 expression with OS and relapse free survival (RFS) in the MYCN-na and MYCN-a subsets of NB patients, and whether RD3 loss is acquired with the IMCT using web-based application (http://r2.amc.nl). We examined the association of acquired RD3 loss with poor prognosis using multiple cohorts of NB patients and cell lines.

## Results

### RD3 loss is associated with poor clinical outcomes in MYCN-na NB

The significance of RD3 rearrangement in NB progression and its potential as the diagnostic or prognostic indicator could be critical for early detection/prediction and for improved targeted therapeutic strategies. Recently, we reported the instrumental role of RD3 in NB pathogenesis and indicated its negative association with disease progression and clinical outcomes. With *in silico* analysis, we examined the correlation of RD3 expression with risk status, prognosis, disease stage, OS, RFS, and event-free survival (EFS) in MYCN-na subset of NB patients. First, low levels of RD3 expression directly correlated with high-risk status (Fig. S1-A). Second, in a cohort of 100 patients, we observed that RD3 loss correlated highly with unfavorable prognosis (Fig. S1-B). Next, in a cohort of 23 patients, the analysis indicated the association of RD3 loss with advanced disease stage (Fig, S1-C). Further, results from a cohort of 47 patients showed significantly less (*P* < 0.037) RD3 expression in stage 4 disease than in stage 1 disease (Fig. S1-D). Additional studies with a cohort of 30 patients validated the sequential loss of RD3 in high-risk stage 4 NB (*vs*. stage 2, *P* < 0.08; Fig. S1-E). With regard to clinical outcomes, low RD3 expression was inversely correlated with OS in a cohort of 72 patients (Fig. S1-F). This negative correlation with RD3 loss was magnified when computed for RFS (Fig. S1-G). Additional studies with a group of 401 MYCN-na patients validated (*P* < 0.059) this inverse correlation of RD3 loss with poor EFS (Fig. S1-H). Conversely, we did not see any significant association of RD3-loss to the OS or RFS in MYCN-a NB subset (Fig. S1-I&J). These data show that there is a significant loss of RD3 in MYCN-na NB patients as the disease progresses. RD3 level is substantially correlated with OS and RFS of patients presenting with MYCN-na NB.

### Regulation of RD3 transcription with IMCT in MYCN-na NB cells

To define changes in RD3 transcription in MYCN-na NB with IMCT, we compared RD3 mRNA levels (ISH, Fig. 1a) in a panel of cell lines derived from stage 4 patients during diagnosis or after IMCT. RD3 ISH revealed measurable basal levels of RD3 mRNA in cell lines (CHLA-42, CHLA-15) derived during diagnosis (Fig. 1b). Compared with these findings, we observed a significant (*P* < 0.001) reduction of RD3 levels in cell lines derived from PD after IMCT. The loss of RD3 transcription remained consistent in all cell lines after IMCT (Fig. 1b). Validating the ISH data, QPCR analysis revealed significantly (*P* < 0.001) lower levels of RD3 mRNA in the cell lines derived after IMCT compared with CHLA-15 and CHLA-42 cells (Fig. 1c). The results are consistent across cell lines and corroborate well with the RD3 ISH data. We observed extensive inter-clone fluctuations of RD3 mRNA levels in CHLA-20 cells measured under both the ISH and QPCR platforms.

Utilizing an *in silico* approach, we investigated the IMCT-associated definitive loss of RD3 mRNA in MYCN-na cell lines. We crisscrossed and compiled RD3 expression in MYCN-na cell lines from multiple independent gene profiling studies submitted by individual investigators. The benefits of such a strategy permit (i) identification and confirmation of the variations in RD3 mRNA levels before or after IMCT; (ii) validation with a third, better platform (RNA sequencing); (iii) representation by the same cell lines across platforms, which rules out equivocal claims, and; (iv) experimental investigations for the proof-of-concept. We investigated expression of RD3 in 15 MYCN-na cell lines, including those derived during diagnosis (NBLS, NB16, NB69) and after IMCT (ACN, GI-ME-N, LA-N-6, NB-1, SK-N-FI, SHEP2, NB-EBc1, SJNB1, SJNB12, COGN549, COGN534, SK-N-SH). The details for these cell lines, including patient gender, age, disease stage, MYCN status, phase of therapy, source of culture, and IMCT, are provided in Table S1. Of these, LA-N-6, SK-N-FI, and NB-EBc1 were also part of the analysis with ISH and QPCR, and hence allowed us to perform inter-platform comparisons. Compared with the three cell lines derived during diagnosis, RNA sequencing data demonstrated a significant reduction of RD3 transcription in all cell lines derived after IMCT (Fig. 1d). RNA sequencing data corroborated well with our ISH and QPCR observations. The RD3 mRNA expression in LA-N-6, SK-N-FI, and NB-EBc1 exhibited near-identical expression across platforms investigated, validating the ISH and QPCR results (Fig. 1). An independent study showed complete loss of RD3 mRNA in SK-N-SH cells surviving cisplatin or doxorubicin treatment (Fig. 1d), a clear demonstration of RD3 transcriptional loss in cells surviving chemotherapeutic drugs.

### RD3 protein loss with IMCT in MYCN-na NB cells

**Figure 2:**
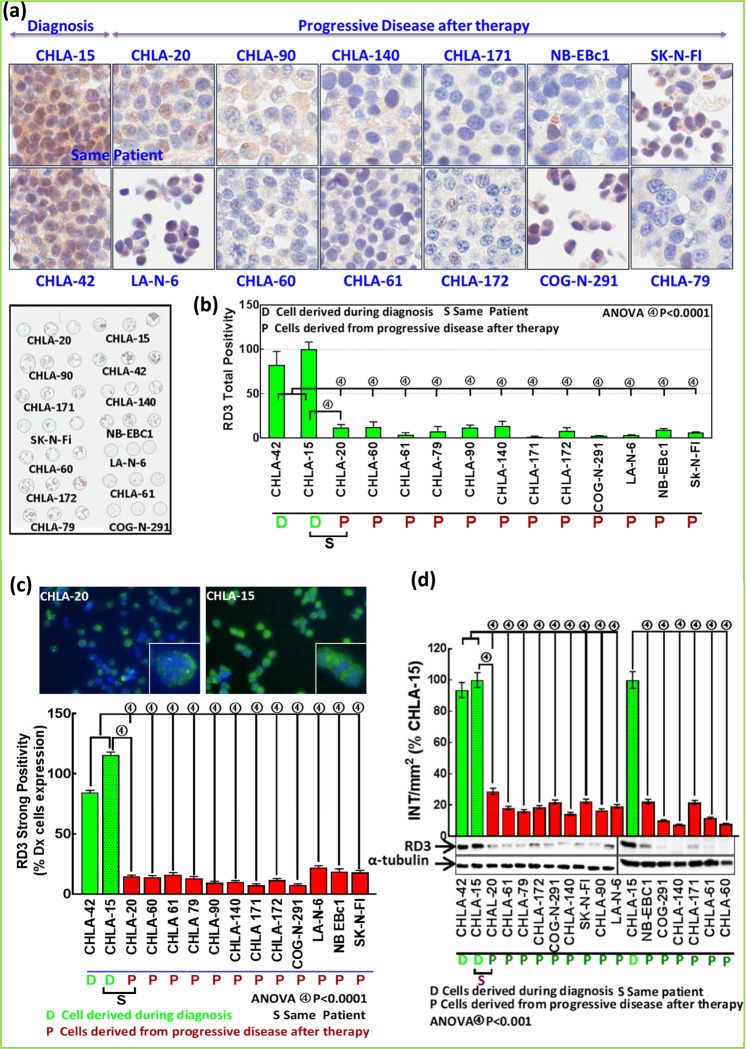
Acquired loss of RD3 protein with IMCT: (**a**) Representative microphotographs of RD3-immunostained MYCN-na NB cells showing expression and localization disparities of RD3 between cell lines derived at diagnosis *vs*. progressive disease (PD). *Lower Panel*. Custom-archived CMA with the panel of 14 MYCN-na NB cell lines. (**b**). Histograms from Aperio CMA image analysis and quantification for RD3 total positivity showing abundant positivity in diagnosis cell lines, but weak and less abundant RD3 positivity in cell lines from PD after IMCT. (**c**) Representative microphotographs from high-content confocal microscopy showing RD3 localization and expression in CHLA-15 (Dx) and CHLA-20 (PD) cells. *Bottom Panel*. Histograms obtained from Columbus quantitative image analysis showing strong and abundant RD3 positivity in two diagnosis-derived cell lines and low abundance and weak positivity of RD3 in 12 PD (after IMCT) cell lines. (**d**) Representative immunoblots and histograms from band intensity analysis showing acquisition of significant RD3 loss in PD cell lines compared with those derived during diagnosis. Full-length blots are presented in Supplementary Fig. S5.

To determine the acquired loss of RD3 protein in MYCN-na NB cells after IMCT, we investigated the localization and expression levels of RD3 in 15 stage 4 MYCN-na cell lines. IHC staining consistency across cell lines was achieved by custom-archived CMA (Fig. 2) and triplicate cores for each cell line, coupled with automated IHC. RD3 IHC revealed abundant positivity in CHLA-15 and CHLA-42 cell lines. Selective RD3 localization was observed in the cytoplasm, perinuclear region, and nucleus (Fig. 2a). We observed no immunoreactivity in CMA-IHC with no primary Ab and IgG controls, and this served as the negative control.

Although RD3 staining was detectable in all cell lines, there was a substantial variance in its localization and expression between cell lines. In CHLA-20, SK-N-FI, CHLA-172, and COG-N-291 cells, we observed weak cytoplasmic staining with less abundant, weak-to-moderate nuclear staining. CHLA-60, CHLA-79, CHLA-61, CHLA-90, CHLA-140, CHLA-171, NB-EBc1, and LA-N-6 exhibited weak cytoplasmic staining with rare weak nuclear positivity (Fig. 2a). Aperio CMA image analysis and quantification revealed a significant (*P* < 0.001) loss of RD3 protein in cell lines derived after IMCT, compared with the cell lines derived during diagnosis (Fig. 2b). RD3 immunofluorescence (IF) analysis corroborated RD3 cytoplasmic, perinuclear, and nuclear localization (Fig. 2c). We observed a strong and abundant RD3 positivity in CHLA-15 and CHLA-42 cells (Fig. 2c). CHLA-20 cells exhibited weak cytoplasmic positivity and moderate nuclear-specific positivity (Fig. 2c). Image analysis and positivity quantification with Columbus revealed low abundance and weak positivity of RD3 in cell lines derived after IMCT (Fig. 2c). Further, group-wise comparison with GraphPad PRISM revealed a consistent and significant (*P* < 0.001) loss of RD3 in cells derived after IMCT, compared with those derived prior to IMCT (Fig. 2c). The localization and expression levels observed with IF analysis corroborated near-identically with the IHC data. Immunoblotting and subsequent band intensity analysis revealed high levels of RD3 protein in CHLA-15 and CHLA-42 cells, while the NB cells derived after IMCT showed very low levels of RD3 (Fig. 2D). Together, these findings illustrated the loss of RD3 in cells derived from PD after IMCT. These results across platforms strongly suggest the acquisition of RD3 loss in MYCN-na NB cells that survive IMCT.

### Acquisition of RD3 loss in PD after IMCT

**Figure 3:**
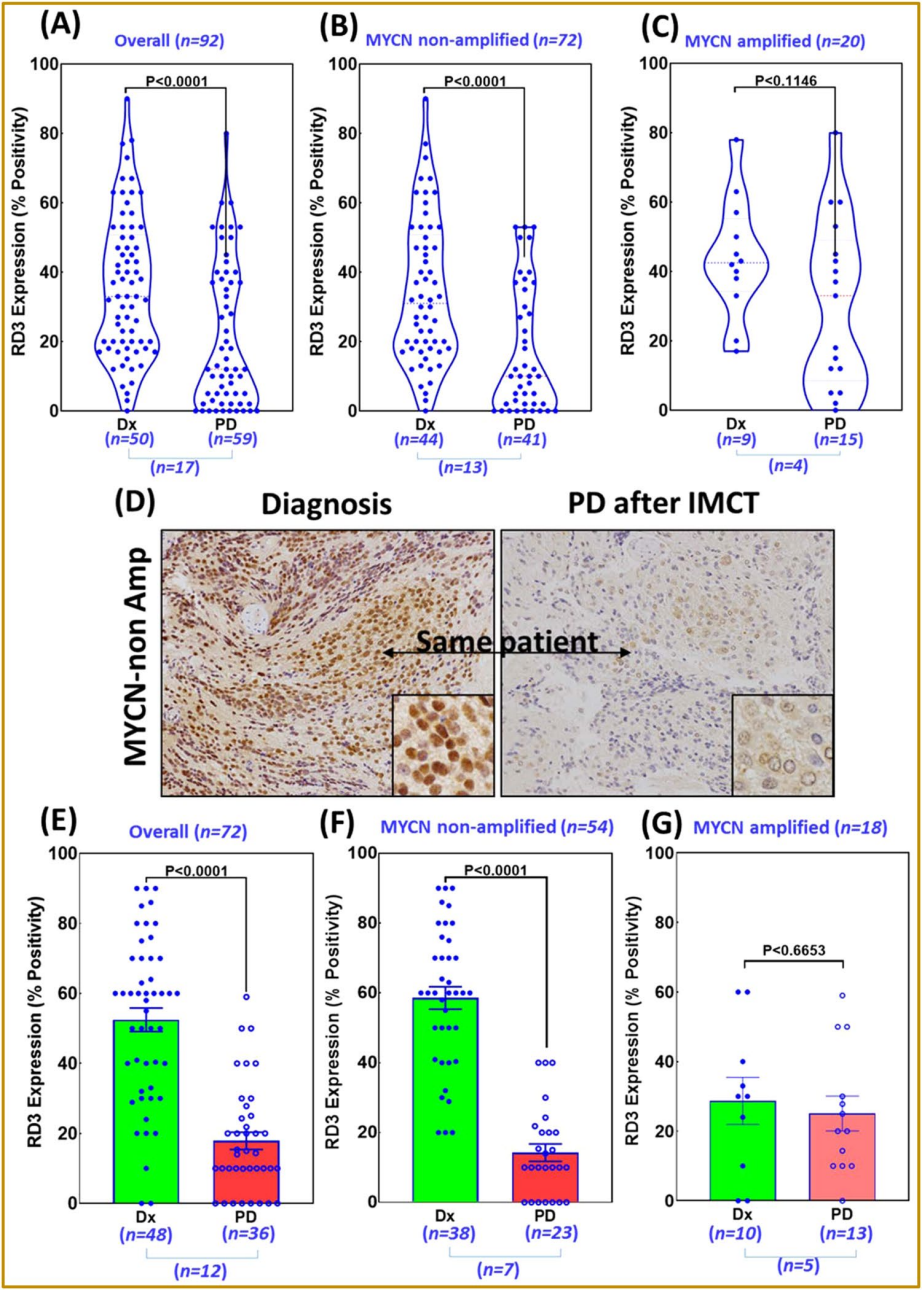
Acquisition of RD3 loss in progressive disease after clinical therapy: (**A**–**C**) RD3 RNAscope analysis showing levels of RD3 mRNA in NB specimens procured during diagnosis (Dx) or at progressive disease after IMCT (PD). Violin plots constructed for (**A**) overall NB patient cohort, (**B**) MYCN-na NB subset and (**C**) MYCN-a NB subset showing fluctuation in RD3 transcription between DX and PD. (**D**) Representative photomicrographs of RD3 immunohistochemistry showing levels of RD3 (20×, insert 60×) in matched (same patient) disease at Dx and PD after IMCT (ii). (**E**–**G**) RD3-IHC analysis showing RD3 protein levels in NB specimens procured during Dx or at PD after IMCT. Histograms of RD3-IHC quantification showing fluctuations of RD3 expression in PD after IMCT (*vs*. disease at Dx) in (**E**) overall NB patient cohort, (**F**) MYCN-na subset and (**G**) MYCN-a subset. ‘*n’* indicates number of cases per group, ‘˽’ indicates *n* number of patients with both Dx and PD specimens. Group-wise comparisons (*t-*test) were made using GraphPad PRISM analysis.

To define the acquired loss of RD3 with IMCT in MYCN-na NB patients, we investigated the fluctuations of RD3 (mRNA and protein) in clinical bio-specimens derived at Dx and at PD after IMCT (Fig. 3). RNA ISH performed in a cohort of 92 patients (Dx, *n* = 50; PD, *n* = 59) revealed a significant (P < 0.0001) loss of RD3 in PD after IMCT compared to Dx (Fig. 3A). The inclusion of 17 cases in this cohort with specimens both from Dx and PD serves as the internal controls. Selectively, the MYCN-na (*n* = 72) subset affirms a significant (P < 0.0001) acquired loss of RD3 in PD after IMCT (*n* = 41) as opposed to Dx (*n* = 44) (Fig. 3B). Conversely, compared to Dx (*n* = 9) we did not see any definitive association of RD3 loss in PD after IMCT (*n* = 15) in MYCN-a subset (Fig. 3C). Assessment of RD3 protein fluctuations between DX and PD after IMCT was performed in 72 cases, with at least 12 cases having specimens both at Dx and PD. RD3-IHC revealed a significant loss of RD3 in specimens derived from PD after IMCT compared with specimens derived at diagnosis, in the same patient (Fig. 3D). Overall, compared to Dx (*n* = 48), we observed a significant RD3-loss in PD after IMCT (*n* = 36) (Fig. 3E). More importantly, MYCN-na subset (*n* = 54) displayed a definitive (P < 0.001) RD3-loss in PD after IMCT (*n* = 23) compared to Dx (*n* = 38) that includes 7 cases with DX/PD specimens (Fig. 3F). Interestingly, MYCN-a subset (*n* = 18) did not reveal any significant RD3-loss in PD (*n* = 13) *vs*. Dx (*n* = 10) regardless of 5 cases with DX/PD specimens (Fig. 3G). Together these data indicate an acquired RD3 loss in PD after IMCT when compared to those from Dx and further imply that this could be more prominent in MYCN-na subset. Both ISH and IHC data are in strong agreement across and further, these observations are in line with our *in vitro* (and *in silico*) RD3 transcription and translation data affirming that RD3 loss is acquired in NB cells that survive IMCT.

### RD3 loss is instrumental in MYCN-na NB evolution

To better underscore the role of RD3 loss in MYCN-na NB pathogenesis/disease evolution, we investigated the associations and effects in *in vitro*, *in vivo*, and *ex vivo* settings. A mouse model of progressive disease was developed using poorly differentiated MYCN-na SK-N-AS cells. Subcutaneous xenografts developed over 30 days in 70–80% of the animals. The xenografts exhibited steady linear growth without metastasis (non-metastatic primary xenograft, NM-PX; Fig. S2-i). However, 20–30% of the mice that received identical clones under similar conditions presented with multiple clinically-mimicking metastatic tumors in the mediastinum and retroperitoneal, pelvic, abdominal, and chest cavities in about 50–60 days (progressive disease [PD]; Fig. S2-ii–v). Development of PD was rapid (Fig. S2-ii) and exceedingly vigorous, and produced 5–8 large (Fig. S2-iii), often multi-lobular (Figure S2-v), viable tumors with well-organized blood supplies. Visually examining the tumor dissemination to vital organs, we observed liver metastasis (Fig. S2-iv). However, histological screening of H&E-stained sections by the pediatric pathologist identified tumor dissemination to the liver, kidney, and lungs (Fig. S3-A). Staining with synaptophysin, the IHC marker for NB^32^, identified tumor dissemination to the liver, lungs, and kidney (Fig. S3-B). The selectivity (neuronal) and localization (selective membrane and cytoplasmic) specificity were validated by including sections from brain and retroperitoneal tumor mass. Synaptophysin staining revealed tumor cell infiltrations in spleen, gut, large intestine, small intestine, and caecum (Fig. S3-B). Metastatic site-derived aggressive cells (SK-MSDACs), which are CSC-like cells derived from metastatic sites, grown *ex vivo* in stem cell medium exhibited organized tumorosphere formation (Fig. S3-vi). Xenotransplantation of MSDACs in mice demonstrated their tumorigenic capacity and reproducibility of PD (Fig. S2-vii). Utilizing the NM-PX and PD mouse models and the MSDACs, we investigated the association of (***a***) transcriptional and/or (***b***) translational loss of RD3 in disease progression, and the driving role of RD3 loss in the pathogenesis of MYCN-na NB progression, (***c***) tumor cell migration, (***d***) invasive potential, and (***e***) tumorosphere formation.

#### Reduced RD3 mRNA levels in PD

**Figure 4:**
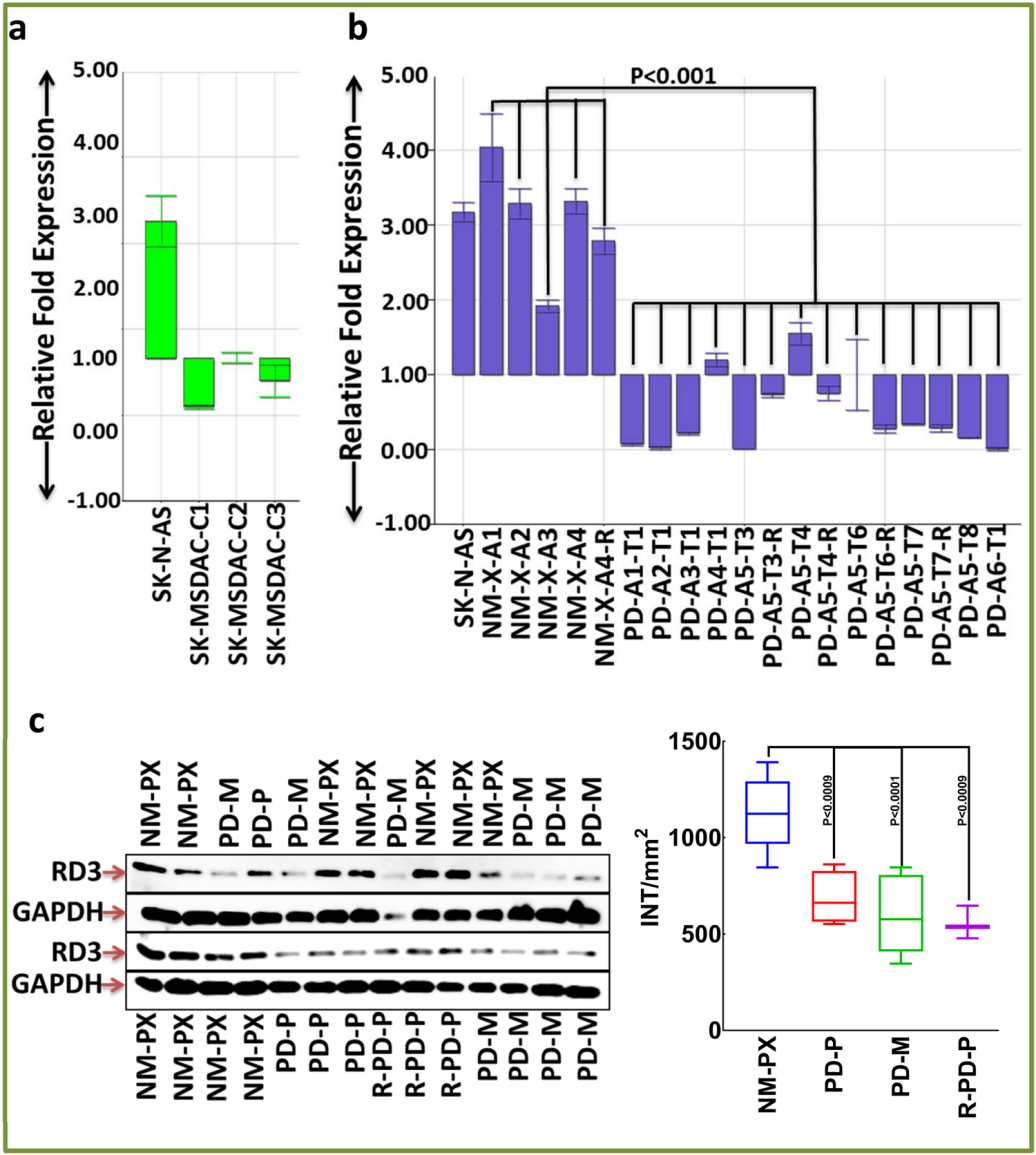
Loss of RD3 in a mouse model of progressive disease (PD)*:* (**a**) Histograms from QPCR analysis showing complete suppression of RD3-mRNA levels in the clones of SK-MSDACs grown *ex vivo* under stem cell culture conditions. (**b**) Histograms from QPCR showing complete loss of RD3 mRNA levels in tumors from primary and metastatic sites of mice with PD (*n* = 6) compared with the primary xenografts from mice without PD (*n* = 6). RD3 mRNA levels were compared between non-metastatic primary xenografts (*n* = 4), metastatic lesions from animals bearing progressive disease (*n* = 6), multi-metastatic lesions from the same animal (*n* = 5), a reproduced non-metastatic xenograft (*n* = 1), and reproduced progressive disease (*n* = 3). Parental SK-N-AS cells are used for expression comparison. (**c**) Immunoblots showing consistent loss of RD3 in tumors from primary (Top panel) and metastatic sites (Bottom panel) of mice with PD compared with primary xenografts in mice without PD. *Side panel*. Band intensity analysis and group-wise comparison showing significant loss of RD3 in PD. Densitometry analyses were performed using Quantity One Image analysis software and were compared using GraphPad PRISM. ***NM***, *non-metastatic*; ***PD***, *progressive disease*; ***A***, *animal*; ***T***, *tumor*; ***X***, *primary xenograft*; ***R***, *reproduced disease*; ***P***, *tumor from primary site*; ***M***, *tumor from metastatic site*. Full-length blots are presented in Supplementary Fig. S5.

RD3 mRNA levels in various clones of MSDACs were examined. We observed a significant reduction of RD3 mRNA levels in all three MSDAC clones investigated compared with parental SK-N-AS cells (Fig. 4a). Next, we explored the alterations in RD3 mRNA in tumor tissues from multiple metastatic sites from different animals and compared the results with those from NM-PX controls. To eliminate any equivocal inference, we included tumor tissues from different animals, different metastatic tumors from the same animal, and different sampling locations from the same tumor. SK-N-AS cells were included in the assay to infer the expression range. Compared with the NM-PX controls, we observed a significant (*P* < 0.001) reduction in RD3 mRNA levels in PD tissues (Fig. 4b). Although we observed inter-animal, inter-tumor (same animal), and intra-tumor variations, transcriptional loss of RD3 in PD metastasized tumors generally displayed consistent reduction over the manifold of NM-PX controls (Fig. 4b).

#### Loss of RD3 protein in PD

**Figure 5:**
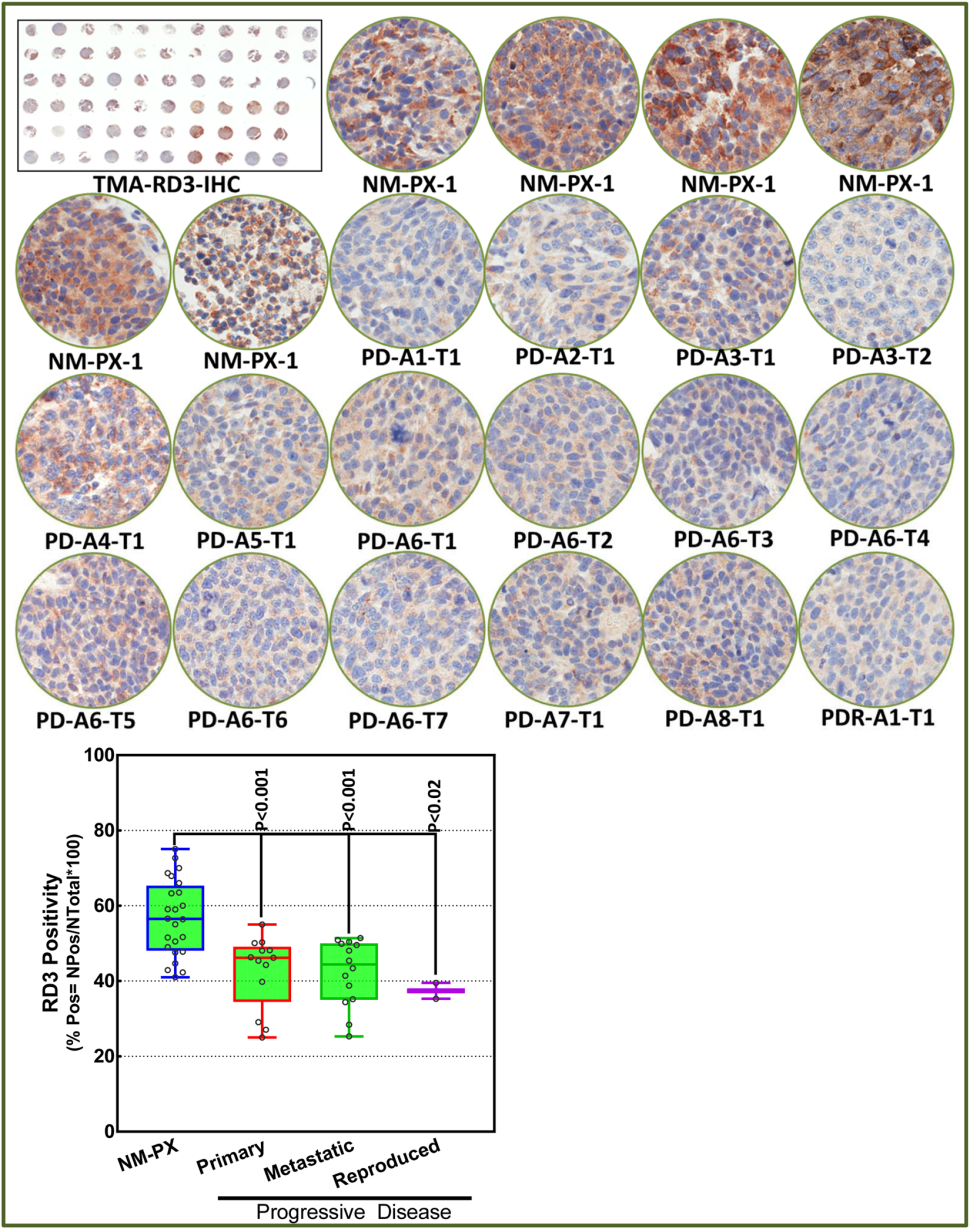
Loss of RD3 in mouse model of progressive disease (PD): (**A**) Thumbnail and constructed images (20×) from custom-archived TMA immunostained for RD3 showing localization and expression levels in primary and metastatic sites of mice with PD compared with the primary xenografts in mice without PD. *Side Panel*. Aperio TMA analysis coupled with group-wise comparison (GraphPad) showing significant loss of RD3 in primary and metastatic site tumors of PD mice and in reproduced disease with MSDACs compared with primary xenografts from mice without PD.

Variations in RD3 protein expression between the primary xenografts from mice with favorable disease, primary and metastatic sites from mice with PD, and primary xenografts from mice with reproduced disease were investigated with immunoblotting. NM-PX controls consistently exhibited measurable baseline RD3 expression. We observed a substantial loss of RD3 in primary and metastatic site tumor tissues from PD animals compared with NM-PX animals (Fig. 4c). Furthermore, primary xenograft with MSDACs exhibited RD3 loss (Fig. 4c). Band intensity analysis revealed (*P* < 0.001) the loss of RD3 in PD when compared with NM-PX animals (Fig. 4c). To further substantiate the RD3 loss in PD, we custom-archived the NM-PX, PD-primary xenografts, PD-metastatic tumors, and reproduced disease with MSDACs in TMA and immunostained for RD3 (Fig. 5). We observed an intense RD3 immunoreactivity in NM-PX animals and a very weak positivity in tissues from primary and metastatic sites of animals with PD (Fig. 5). TMA quantitation demonstrated a significant (*P* < 0.001) loss of RD3 in tumor tissues from primary and metastatic sites of mice with PD (Fig. 5). Consistently, we observed weak RD3 localization in MSDAC-derived (reproduced) disease. The IHC data concur with the immunoblotting data. Together, the protein expression data corroborate the mRNA data and affirm the significant transcriptional and translational loss of RD3 in MYCN-na progressive NB.

#### RD3 regulates NB cell migration

**Figure 6:**
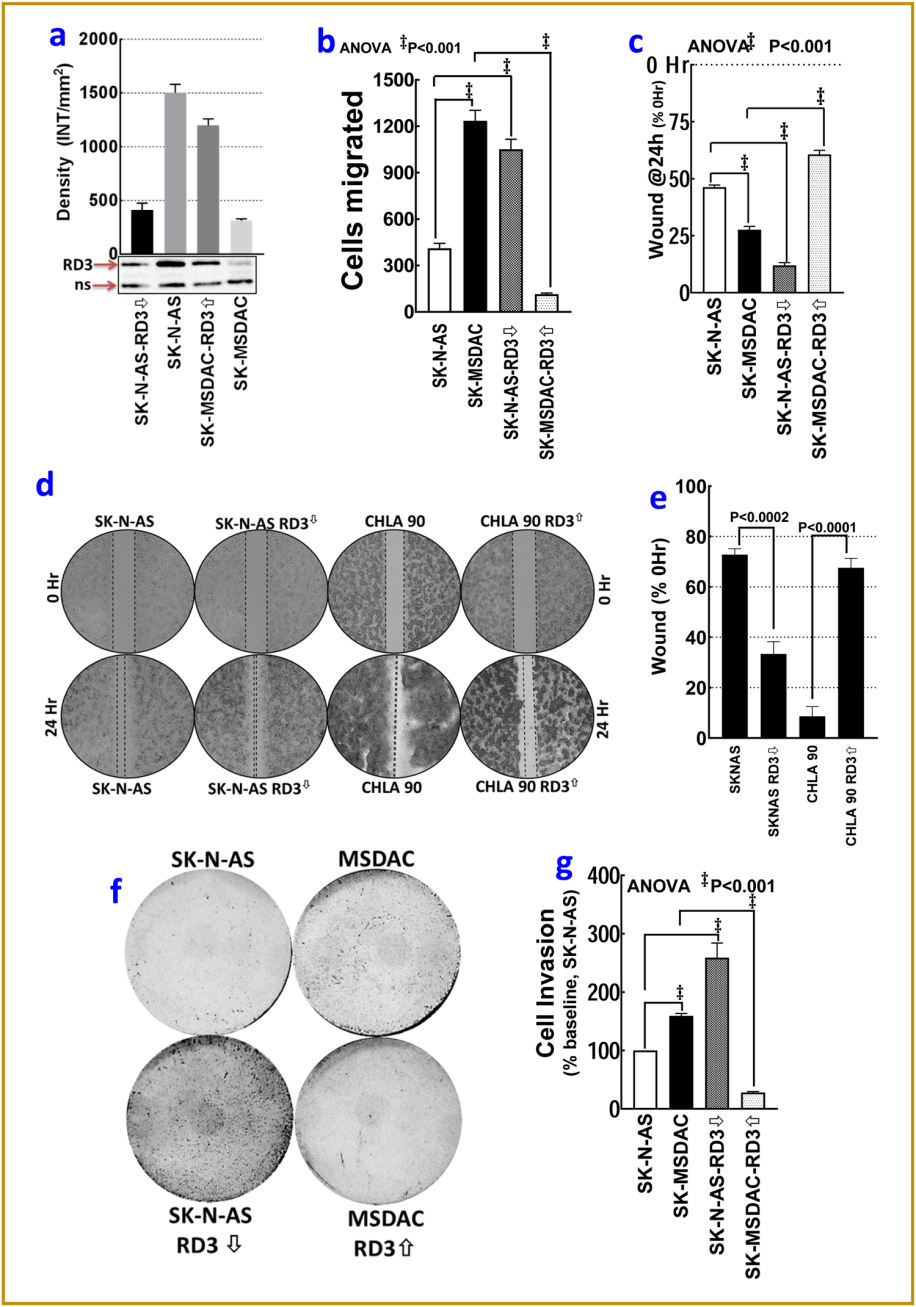
RD3 loss in the pathogenesis of progressive disease (PD): (**a**) Immunoblots showing the re-expression of RD3 in SK-MSDAC and silencing of RD3 in SK-N-AS cells. Full-length blots are presented in Supplementary Fig. S5. (**b**) Histograms from ORIS cell migration analysis exhibiting robust cell migration of SK-MSDACs and RD3-silenced SK-N-AS (*vs*. parental SK-N-AS). Re-expression of RD3 in SK-MSDACs completely regulated the heightened migration capacity of SK-MSDACs. (**c**) Histograms of scratch wound gap measurements (mean and *SD*) showing the cell migration patterns of SK-MSDACs, with and without RD3 re-expression, and parental SK-N-AS cells, with and without RD3 silencing under proliferation controlled conditions, examined at 24 h after wound initiation. SK-MSDACs exhibit robust cell migrations with significant wound closure after 24 h, while re-expression of RD3 in MSDACs significantly inhibited their migration. Silencing RD3 in SK-N-AS cells consistently increased their migration with significant wound closure. (**d**) Microphotographs and (**e**) histograms of scratch wound gap measurements (mean and *SD*) showing the cell migration patterns of stage 4 MYCN-na patient derived PD cell-line CHLA-90 (that displayed high RD3-loss), with and without RD3 re-expression. SK-N-AS cells, with and without RD3 silencing were used as assay controls. CHLA-90 exhibit robust cell migrations with significant wound closure after 24 h, while re-expression of RD3 significantly inhibited their migration. (**f**) Photographs of Matrigel invasion assay showing robust invasion of SK-MSDACs, completely alleviated invasion in RD3-re-expressed SK-MSDACs, and a profound increase in the invasive potential of RD3-silenced SK-N-AS cells. (**g**) Histograms of Matrigel-invaded cells (mean and *SD*) showing complete inhibition of SK-MSDACs invasion potential with RD3 re-expression and a significant increase in invasiveness of RD3-silenced SK-N-AS cells.

To define the role of RD3 loss in the pathogenesis of the disease, we compared the cell migration patterns in parental SK-N-AS cells, RD3-less SK-MSDACs, RD3-muted SK-N-AS cells and in RD3-re-expressed SK-MSDACs (Fig. 6a). Since MSDACs and RD3-silenced SK-N-AS cells are loosely adherent and display sphere-like growth patterns at confluence, a scratch-wound assay could produce equivocal outcomes. Hence, we assessed cell migration with two independent platforms (Fig. 6b–e). Compared with RD3-expressing SK-N-AS cells, under proliferation-controlled conditions, cell migration analysis showed a profound migration of SK-MSDACs (Fig. 6b). Conversely, re-expression of RD3 in MSDACs resulted in reduced migration. Muting RD3 in SK-N-AS cells significantly increased their migration capabilities (Fig. 6b). Substantiating these results, assessment of wound closure with a conventional scratch-wound assay revealed (i) significant wound closure by SK-MSDACs (compared with SK-N-AS), (ii) reduced wound closure in RD3 re-expressed SK-MSDACs (compared with SK-MSDACs), and (iii) heightened wound closure in RD3-muted SK-N-AS cells compared with SK-N-AS cells (Fig. 6c). Further to corroborate the role of RD3 in the regulation of NB cell migration, we examined the influence of RD3 reinforcement in RD3 null patient derived CHLA-90 cells. For this wound closure was compared between CHLA-90 cells with and without ectopic expression of RD3. SK-N-AS with and without RD3 silencing is used in parallel as assay control. Compared to SK-N-AS controls, CHLA-90 cells showed significant would closure. Conversely, reinforcement of RD3 in CHLA-90 cells significantly inhibited their migrator capabilities (Fig. 6d,e). These data strongly suggest that RD3 regulates NB cell migration, and the loss of RD3 in PD leads to an increased rate of cell migration.

#### RD3 regulates NB cell invasion capabilities

Matrigel invasion analysis displayed a robust metastatic potential of MSDACs with a heightened number of invading cells (Fig. 6f). Compared with SK-N-AS cells, this increase in invasiveness of SK-MSDACs was statistically significant (Fig. 6g). While re-expressing RD3 significantly (*P* < 0.001) delimited SK-MSDACs invasion potential, silencing RD3 in SK-N-AS cells resulted in high invasiveness (Fig. 6f,g). These findings illustrate the role of RD3 in regulating tumor cell invasion.

#### RD3 regulates tumorosphere formation

LDTA analysis with SK-N-AS cells with/without RD3 silencing and SK-MSDACs with/without RD3 re-expression demonstrated that RD3 regulates the formation of tumorospheres in stem cell culture conditions. The time-lapse images (18 h) of cells plated in serum-free stem cell medium stained with DiI were stitched as video (Video S1). SK-N-AS cells exhibited monolayer cell spreading without any organized tumorosphere formation (Video S1 *Top left*). Silencing RD3 in SK-N-AS cells resulted in the formation of organized tumorospheres and defied any monolayer cell spreading (Video S1 *Bottom left*). SK-MSDACs exhibited organized tumorosphere formation without any monolayer cell spreading (Video S1 *Top right*). However, re-expressing RD3 in SK-MSDACs abrogated organized tumorosphere formation (Video S1 *Bottom left*). We observed no monolayer cell spreading in these RD3 re-expressed SK-MSDACs.

### Experimental studies affirm IMCT-acquired RD3 loss in MYCN-na NB

To further substantiate the acquisition of RD3 loss, we cross-examined publicly available data from experimental NB studies, with a particular focus on the MYCN-na NB. Treatment with ALK inhibitor TAE-684 showed significant loss of RD3 in surviving MYCN-na CL-BGA and SK-N-SH cell lines (Fig. S4A&B). In another study, acquisition of RD3-loss was evident in the TAE-684-resistant MYCN-na SY5Y cells when compared with the parental TAE-684-sensitive cells (Fig. S4-C). Significant loss of RD3 was observed in cells that survived treatment with IBET-726, the BET bromodomain inhibitor (Fig. S4-D). SY5Y cells that survived THZ1 (CDK7 inhibitor) treatment exhibited marked RD3 reduction compared with vehicle-treated cells (Fig. S4-E). H10H5 (an IGF-1R antibody) treatment of mice bearing SK-N-AS-derived xenografts resulted in significant RD3 loss (Fig. S4-F). These data illustrated acquired RD3 loss in NB cells that survived therapy.

## Discussion

The response rate for high-risk PD remains low. This poor result is attributable to disease evolution with constant and adaptive genetic and molecular rearrangements. Thus, it is pertinent to identify genetic determinants that evolve in response to IMCT that could mediate therapy resistance and lead to disease recurrence. Evidence from our earlier studies defined the instrumental role of RD3 loss in PD pathogenesis^29^. The results presented in the current study, for the first time, identified the acquired loss of RD3 with IMCT. Acquisition of RD3 loss with IMCT was observed at the transcriptional and translational levels. This observation in a panel of cell lines derived from patients with stage 4 disease before and after IMCT not only recognizes the acquired response, but indicates its critical role in MYCN-na disease evolution. More importantly, our direct analysis using a validation cohort of MYCN-na NB patients and comprising bio-specimens acquired during Dx and at PD after IMCT affirmed the acquisition of RD3 loss in PD after IMCT. Acquired RD3 loss observed with IMCT remained consistent within and between patients. Further, our crisscross analysis of independent *in silico* RNA seq/microarray data for a dissimilar set of 15 cell lines strongly corroborated our finding that RD3 is significantly lost in cells that survive IMCT.

RD3, the protein abundantly localized in the retina, has defined roles in photoreceptor cell survival^33^. Researchers have extensively documented the functional relevance of RD3 loss inflicted by genetic defect-driven production of less stable truncated protein and subsequent degradation in the orchestration of retinal degeneration^34–38^. However, beyond retinal localization and functions in retinal degeneration, the physiognomy and functions of RD3 are unknown. We were the first to define the distribution of constitutive RD3 expression and localization in healthy human tissues^28^. Although researchers reported RD3 co-localization with the tumor suppressor PML^34^, the functional significance of RD3 in cancer biology is unknown. Our recent study recognizing the loss of RD3 in high-risk NB and its association with advanced disease stage and poor clinical outcomes was the first of its kind to signify the functional relevance of RD3 in NB and beyond^29^. Although RD3 could be regulated by MYCN binding to the promoter^39^, a number of independent studies revealed similar RD3 expression levels in MYCN-na patient cohorts. Here we show that RD3 loss is associated with advanced disease stage, poor prognosis, and worse clinical outcomes in the MYCN-na subset of patients (Fig. S1). To our knowledge, the results presented here for the first time identified the accumulation of RD3 loss in MYCN-na NB cells that survive IMCT.

The criticality of this protein in disease progression/evolution in MYCN-na NB has thus far gone unrecognized. Our MYCN-na subset-focused data mining revealed significant loss of RD3 in cell lines derived during PD after IMCT, when compared with those derived during diagnosis. The results from our *in vivo* studies demonstrated a significant transcriptional and translational loss of RD3 in MSDACs and in metastatic tumors compared with parental SK-N-AS cell and primary xenografts, respectively. Further, our targeted gene silencing/expressing studies defined the critical driving role of RD3 loss in PD pathogenesis. Performing data mining with independent experimental NB studies by other investigators, we observed that MYCN-na NB cells surviving therapeutic agents exhibited significant loss of RD3. These studies are in conceptual agreement with our experimental observations, strongly affirm the ongoing acquisition of RD3 loss in the IMCT surviving MYCN-na NB cells, and indicate its influential role in disease progression.

Interestingly, the results presented here demonstrate RD3 loss at the transcription and translational levels in cells surviving after IMCT. The *in vitro*, *in vivo*, and *ex vivo* studies affirm such a conclusion, indicating that the inflicted response could be beyond any protein modifications, including translational or post-translational events (e.g., phosphorylation, ubiquitination). Loss of RD3 transcription indicates an induced deregulation in RD3 *de novo* synthesis in cells that survive IMCT. Although the exact mechanism of RD3 transcriptional regulation is unclear, we speculate that the epigenetic rearrangements may be one such mechanism

The authors acknowledge the study’s limitations, which include: (i) the culture conditions associated with, or that inflicted, response; (ii) clonal selection and clone-dependent response; (iii) limitations in the number and diversity of cell populations analyzed; and (iv) preclinical experimental mouse models. However, the data from the validation cohort of NB patients, *in vitro* screening, and *in silico* data mining across cell lines, as well as experimental NB studies by others, are in agreement and conceptually affirm the acquired RD3 loss with IMCT in surviving cells. More in-depth preclinical and clinical studies to define the acquired loss, the underlying mechanism, and the criticality of its reinforcement for improved therapeutic strategies are currently under investigation.

## Conclusions

Overall, the results indicated that (i) loss of RD3 is associated with advanced disease stage and poor clinical outcomes in the N-MYC non-amplified subset; (ii) RD3 loss is an acquired process in cells that survive IMCT; and (iii) loss of RD3 has functional relevance in the pathogenesis of progressive disease. More importantly, the results demonstrate that the inflicted RD3 loss is the deregulation in RD3 transcriptional machinery regulating its *de novo* synthesis. Together, the results presented here allow us define the molecular rearrangements in residual cells and pave the way for studies that could help identify a therapeutic target or improved strategy for the better treatment of children with progressive NB.

### Ethical approval

All animal experiments conformed to American Physiological Society standards for animal care and were approved by our University of Oklahoma Health Sciences Center Institutional animal care and use committee (Protocol number 17-035-HCG-H). All animal experiments were performed in strict accordance with institutional guidelines on the handling of laboratory animals.

### Consent for publication

All authors give consent for the publication of the manuscript in the Scientific Reports.

## Supplementary information

Supplementary Data file 1

Supplementary Video 1

## Data Availability

All data generated or analyzed during this study are included in this published article [and its supplementary information files].
