# Supplementary material for: De novo regulation of RD3 synthesis in residual neuroblastoma cells after intensive multi-modal clinical therapy harmonizes disease evolution
Source: Sci Rep. 2019 Aug 13;9:11766. doi: 10.1038/s41598-019-48034-2 (PMC6692366; doi:10.1038/s41598-019-48034-2)
Supplement: Supplementary file 1 — Supplementary Data file 1 [file 41598_2019_48034_MOESM1_ESM.docx]

De novo regulation of RD3 synthesis in residual neuroblastoma cells after intensive multi-modal clinical therapy harmonizes disease evolution

Dineshbabu Somasundaram^1#^, Karthikeyan Subramanian^1#^, Sheeja Aravindan^2#^, Zhongxin Yu^3^, Mohan Natarajan^4^, Terence Herman^1,2^, and Natarajan Aravindan^1,3^


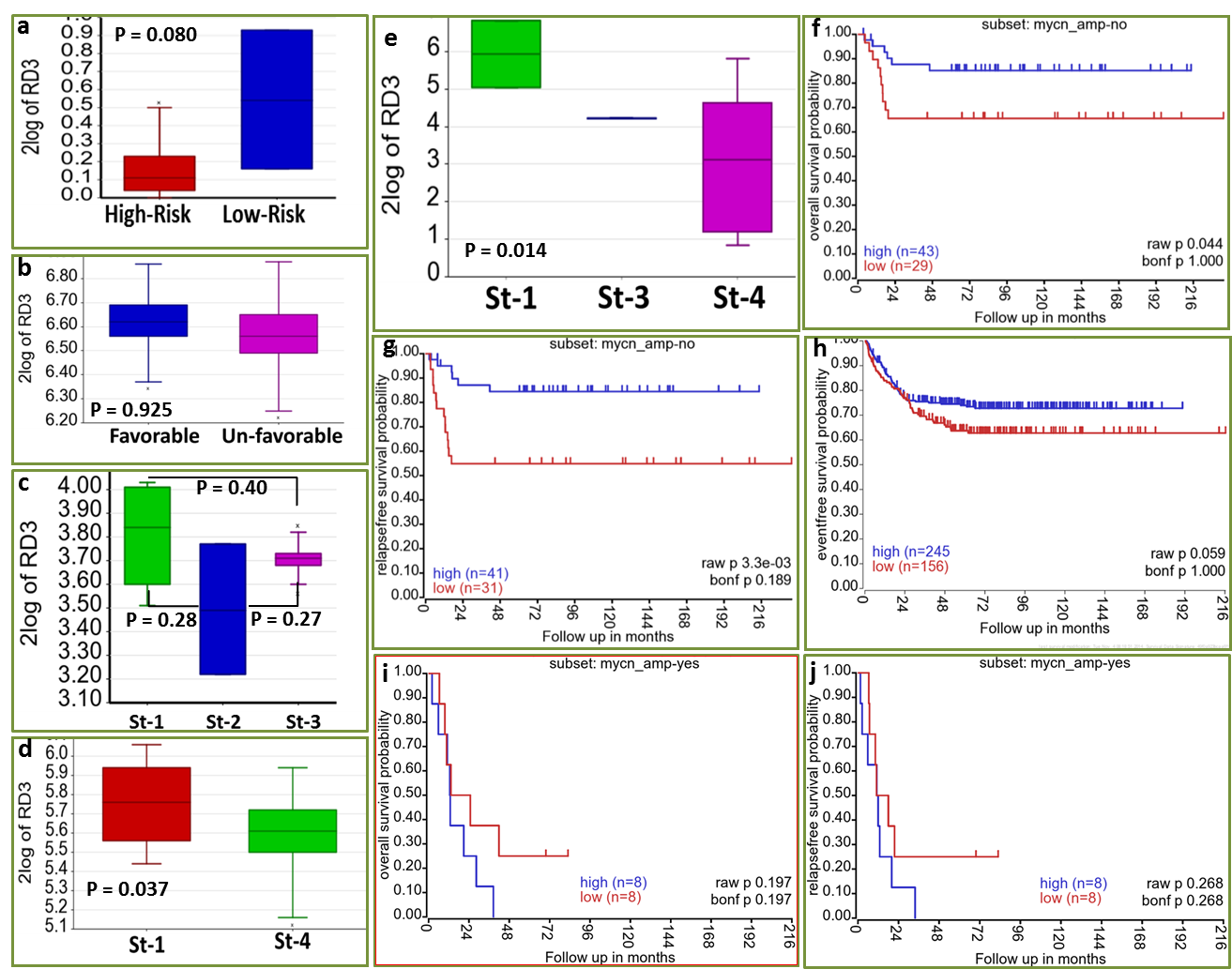


**Figure S1:** RD3 loss is associated with poor clinical outcomes in MYCN non-amplified neuroblastoma (NB): Association of RD3 with (**a**) risk status (*n*=161, NCI-Targett161) and (**b**) prognosis (*n*=100, GSE19274) of MYCN non-amplified NB patients. RD3 loss was correlated with advanced disease stages in cohorts of (**c**) 23 patients (GSE54720), (**d**) 47 patients (GSE27608), and (**e**) 30 patients (GSE13136). Kaplan-Meier curves showing (**f**) decreased overall survival and (**g**) pronounced decrease of relapse-free survival in a cohort of 72 MYCN non-amplified patients (GSE16476) with low RD3 expression compared with high RD3 expression. Kaplan-Meier curve showing a profound decrease in (**h**) event-free survival in MYCN non-amplified patients (*n*=401, GSE62564) with low RD3 expression vs. high RD3 expression. Kaplan-Meier curves showing no significant association of RD3 loss to the (**i**) overall survival and (**j**) relapse free survival in MYCN amplified patients (GSE16476).


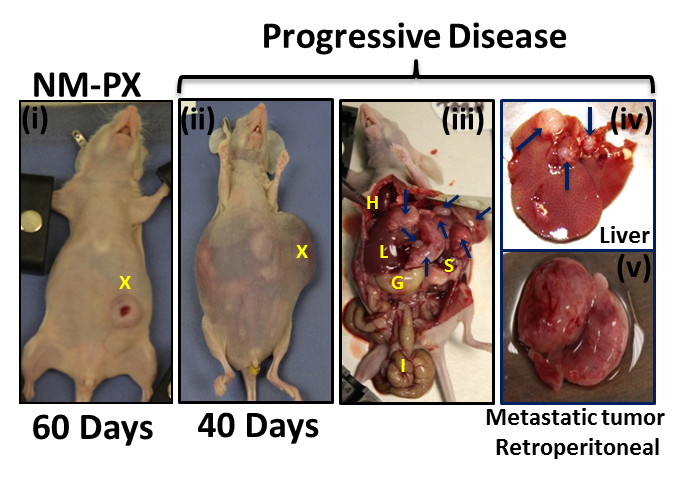


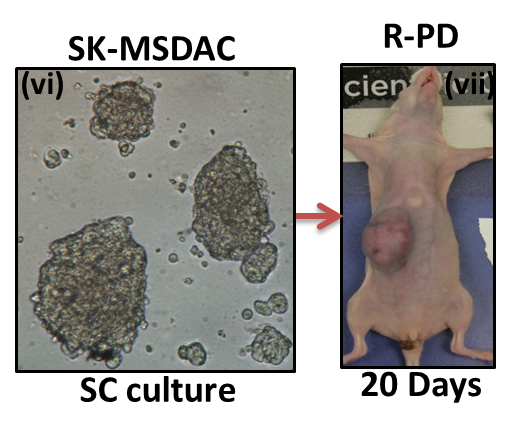


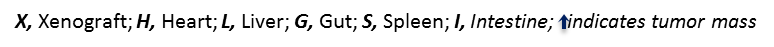


**Figure S2. *Mouse model of MYCN-na progressive disease (PD)***: Representative photographs of human SK-N-AS cells showing that MYCN-na PD developed. (i) Mice showing primary xenografts 60 days after s.c. injection of 5x10^6^ SK-N-AS cells suspended in Matrigel, without metastasis. (ii) Mice showing PD. (iii) Mice with PD showing anatomical locations of the metastatic tumors. (iv) Livers from mice with PD, showing liver metastasis. (v) Photograph showing retroperitoneal tumor mass from a mouse with PD. (vi) Microphotograph (100x magnification) showing formation of well-organized tumorospheres under stem cell culture conditions by the metastatic site-derived aggressive cells (MSDACs) *ex vivo*. (vii) Mice showing the tumorigenic (xenograft development within 20 days) capacity of MSDACs.


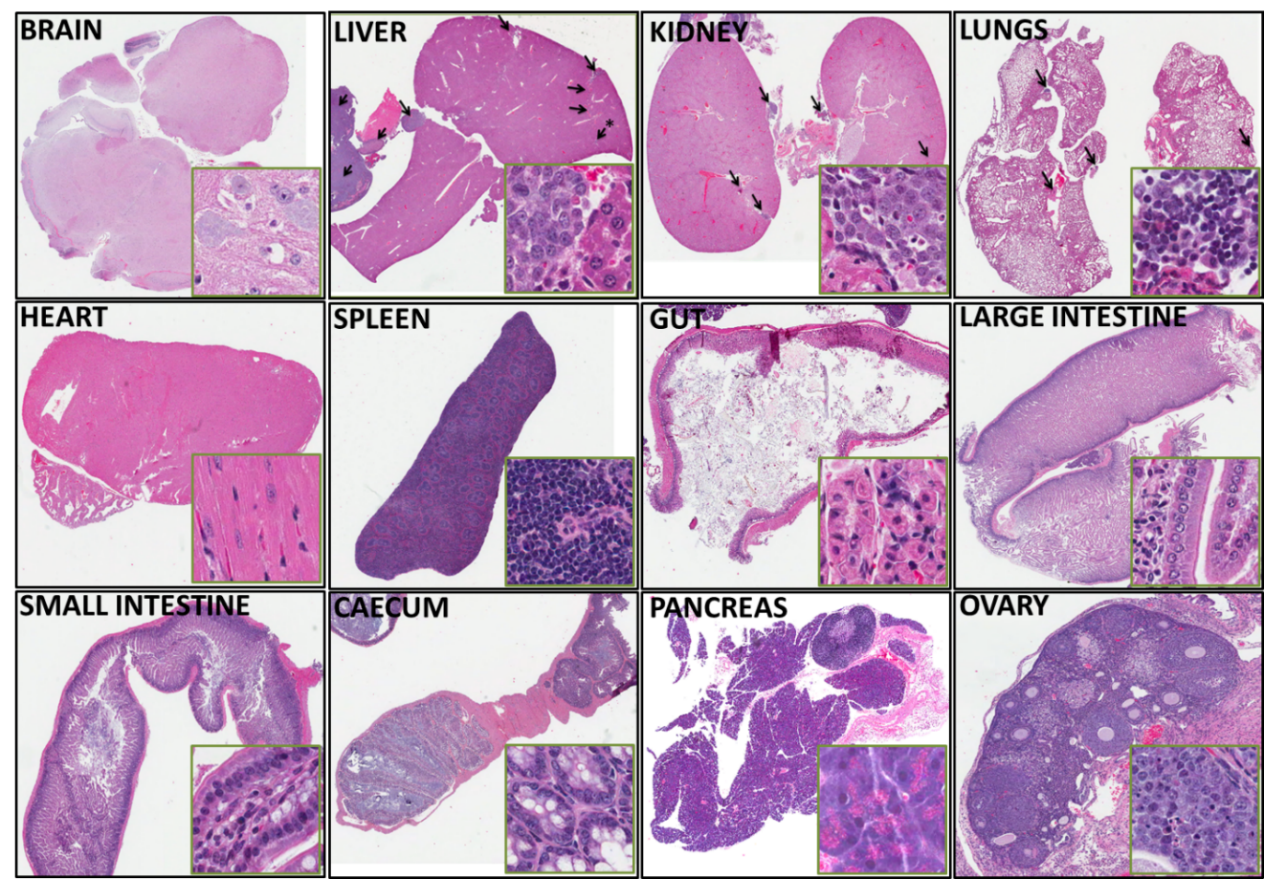

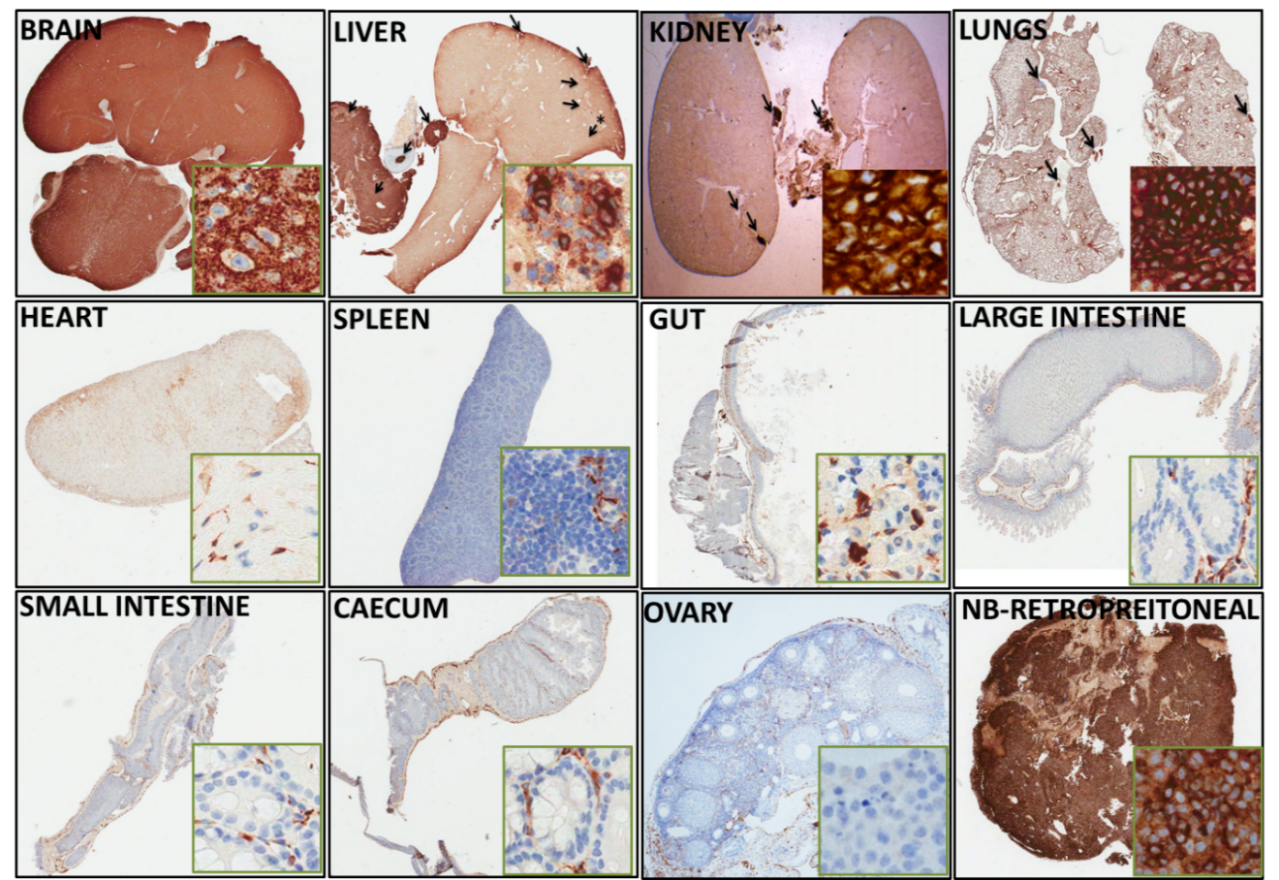


**(B)**

**(A)**

**Figure S3: *Dissemination of neuroblastoma to the vital organs in progressive disease (PD):*** Dissemination of tumor to the vital organs in mice with PD was investigated with **(A)** histological screening of H&E-stained sections from brain, kidney, liver, lungs, heart, spleen, gut, large intestine, small intestine, caecum, pancreas, and ovary by the pediatric pathologist and **(B)** staining with synaptophysin, the IHC marker for neuroblastoma. Histopathological analysis and synaptophysin staining identified tumor dissemination to liver, kidney, and lungs, and tumor cell infiltrations in spleen, gut, large intestine, small intestine, and caecum (indicated by arrowheads).


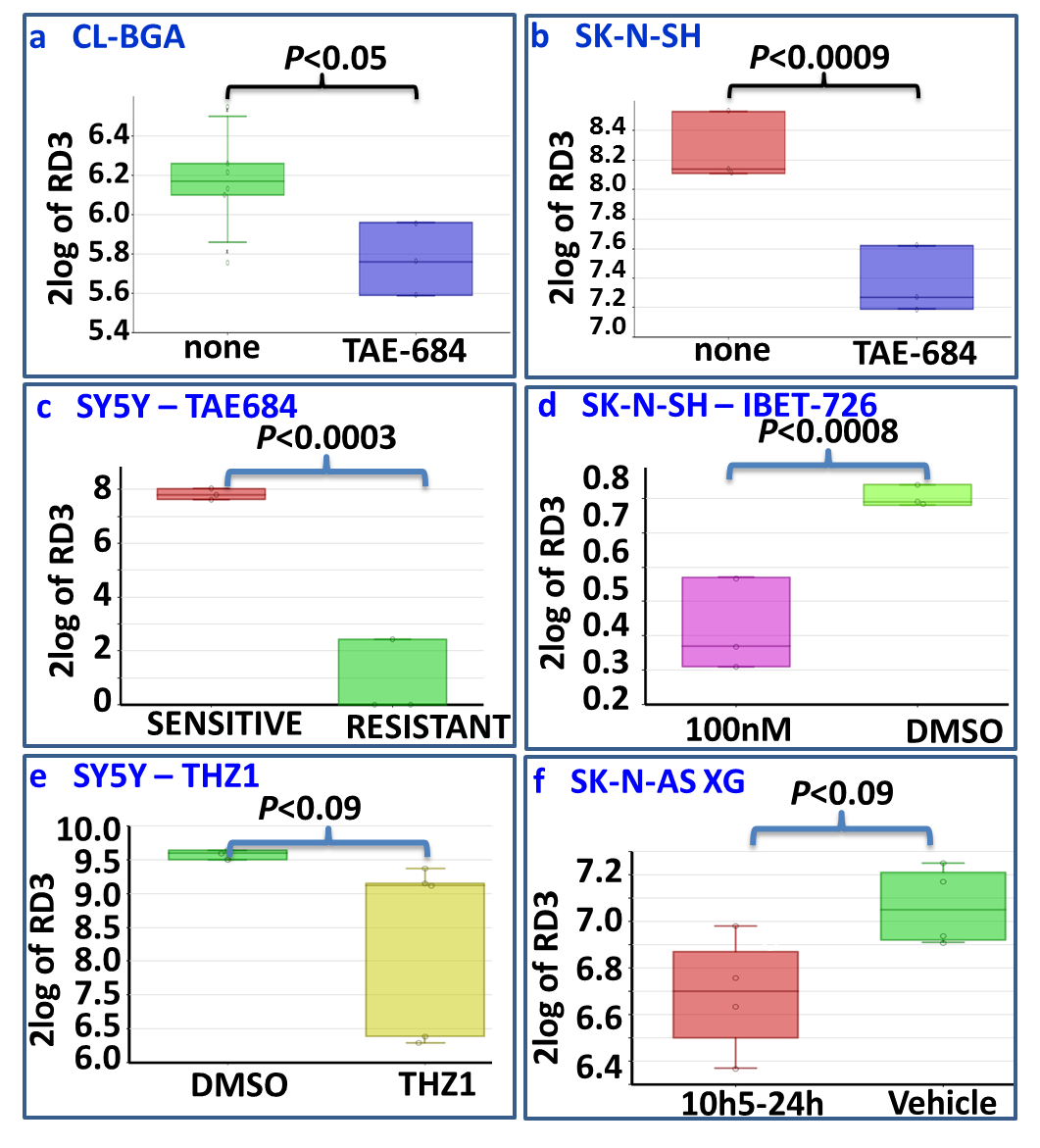


**Figure S4. *Acquired RD3 loss in experimental MYCN-na neuroblastoma:*** Box whiskers plot with circles obtained from independent experimental neuroblastoma studies (<http://r2.amc.nl>) showing acquisition of RD3 loss in resistant cells surviving therapeutic agents. The analysis was focused on the MYCN-na neuroblastoma subset. Treatment with ALK inhibitor TAE-684 showed significant loss of RD3 in surviving MYCN-na **(A)** CL-BGA, **(B)** SK-N-SH, and **(C)** SY5Y cells. Independent studies showing acquired loss of RD3 in cells that survive **(D)** IBET-726, the BET bromodomain inhibitor, and (D) THZ1, a CDK7 inhibitor. **(F)** Study showing acquired RD3 loss in the residual xenografts (SK-N-AS derived) in mice treated with H10H5, an IGF-1R antibody.

**Table S1:** Patient-derived MYCN-na human neuroblastoma cell lines included in the study.

| **Cell line** | **Gen** | **Age** | **Stage** | **MYCN** | **Phase of Therapy** | **Source** | **Therapy** | **PS/IS** |
| --- | --- | --- | --- | --- | --- | --- | --- | --- |
| CHLA-15 | F | 18m | 4 | na | Diagnosis | Primary Tumor | None | PS |
| CHLA-42 |  | 13m | 4 | na | Diagnosis | Bone marrow | None | PS |
| NBL-S | M | 42m | 3 | na | Diagnosis | Adrenal | None | IS |
| NB 16 | F | 35m | 4 | na | Diagnosis | Bone marrow | None | IS |
| NB 69 | M | 28m | 3 | na | Diagnosis | Adrenal | None | IS |
| CHLA-20 | F | 24m | 4 | na | Post-Chemotherapy  (Progressive Disease) | Primary tumor | Cisplatin, cyclophosphamide, doxorubicin, teniposide | PS |
| CHLA-90 | M | 102m | 4 | na | Post-Chemotherapy (Progressive Disease)  Post bone marrow (BM) transplant | Bone marrow | Cisplatin, cyclophosphamide, doxorubicin, teniposide, etoposide, melphalan, Whole body IR | PS |
| CHLA-140 | M |  | 4 | na | Post-Diagnosis  (Progressive Disease) | Bone marrow | Carboplatin, Cisplatin; Doxorubicin, Etoposide, melphalan | PS |
| CHLA-171 | M | 101m | 4 | na | Post- Chemotherapy (Progressive Disease)  post mortem | Peripheral blood | Carboplatin; Etoposide; L-PAM, melphalan | PS |
| NB-EBc1 | M | 36m | 4 | na | Post- Chemotherapy (Progressive Disease) | Retroperitoneal mass | cyclophosphamide, doxorubicin, cisplatin, teniposide | PS/IS |
| SK-N-FI | M | 11Y |  | na | Post- Chemotherapy (Progressive Disease) | Bone marrow |  | PS/IS |
| LA-N-6 | M | 60m | 4 | na | Post-Chemotherapy (Progressive Disease) | Bone marrow | Cyclophosphamide, teniposide, doxorubicin, cisplatin; doxorubicin, etoposide, vincristine, dacarbazine | PS/IS |
| SMS-LHN | M | 24m | 4 | na | Post-Chemotherapy (Progressive Disease) | Solid tumor (femur) | Cyclophosphamide, doxorubicin | PS |
| CHLA-60 | M | 168m | 4 | na | Progressive Disease  (post mortem) | Bone marrow |  | PS |
| CHLA-61 | M | 168m | 4 | na | Progressive Disease  (post mortem) | Blood |  | PS |
| CHLA-172 | M |  | 4 | na | Progressive disease  Post BM transplant | Bone marrow | myeloablative therapy  IR | PS |
| COG-N-291 |  |  |  | na | Progressive Disease | Bone marrow |  | PS |
| CHLA-79 |  | 24m |  | na | Progressive disease  Post myeloablative therapy  Post BM transplant |  | Cyclophosphamide, melphalan, doxorubicin,cisplatin; doxorubicin, etoposide, vincristine, dacarbazine, Whole body IR | PS |
| ACN | M | 39m | 4 | na | Post-therapy | Bone marrow |  | IS |
| GI-ME-N | F | 24m | 4 | na | Post-therapy | Bone marrow |  | IS |
| NB 1 | M | 27m |  | na | Post-therapy | Lymph node |  | IS |
| SHEP 2 | F |  |  | na | Post-therapy | Bone marrow |  | IS |
| SJNB1 | M | 30m | 4 | na | Post-therapy | Adrenal |  | IS |
| SJNB 12 | F | 10m | 3 | na | Post-therapy | Abdomen |  | IS |
| COGN549 |  |  |  | na | Post-therapy |  |  |  |
| COGN534 |  |  |  | na | Post-therapy |  |  |  |
|  |  |  |  |  |  |  |  |  |

**na = not amplified; PS = present study; IS = in silico; m = months; Gen = Gender; M = male; F = Female**


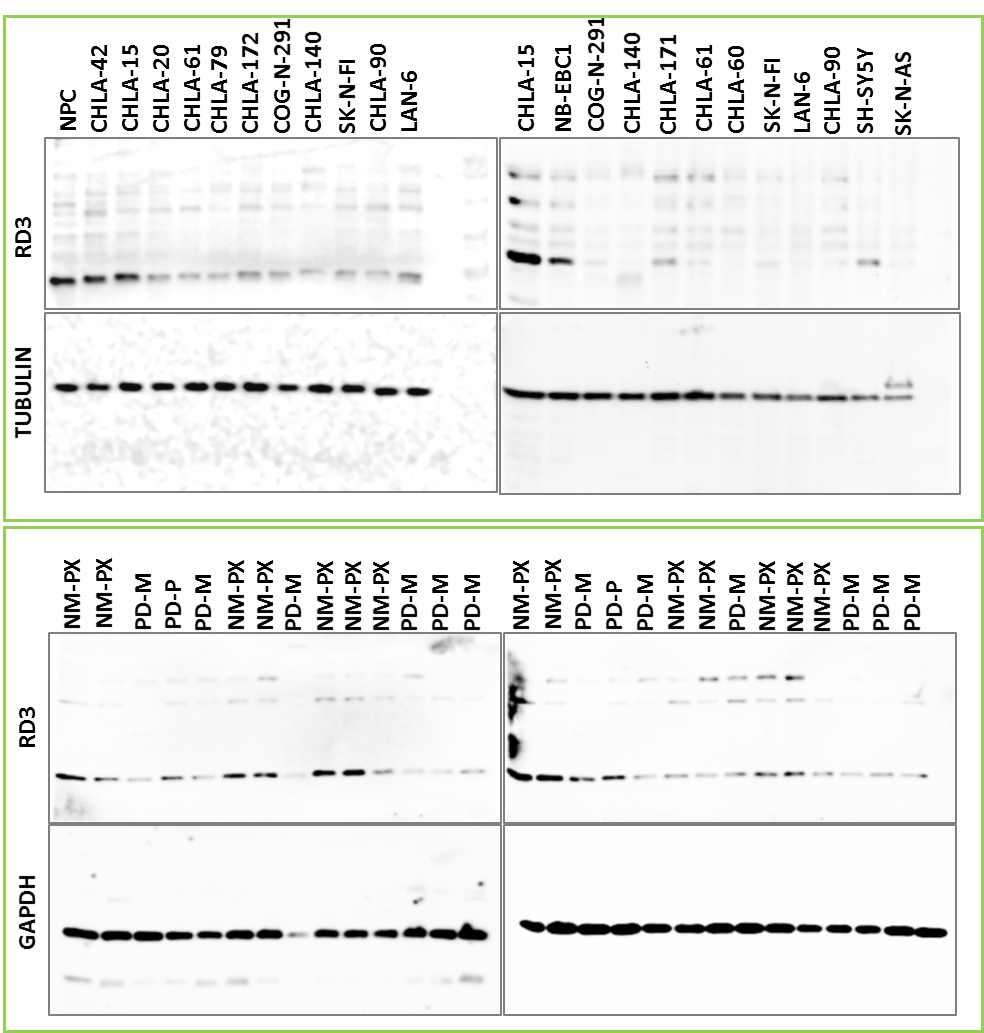


**Figure 3b**

**Figure 4c**

**Figure 4c**

**Figure 3b**


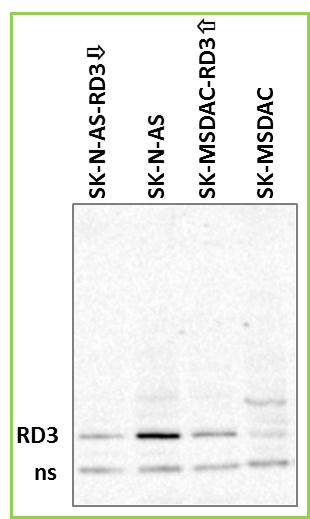


**Figure 6a**

**Figure 6a**

**Figure S5:** Full-length blots for the figures presented in *Figure 3b*, *Figure 4c*, and *Figure 6a*.
